# Construction and Evaluation of a Clinical Prediction Scoring System for Positive Cervical Margins Under Colposcopy

**DOI:** 10.3389/fmed.2022.807849

**Published:** 2022-02-28

**Authors:** Meiling Zhu, Mingyue Yu, Zhengzheng Chen, Weidong Zhao

**Affiliations:** ^1^Department of Obstetrics and Gynecology, Provincial Hospital Affiliated to Anhui Medical University, Hefei, China; ^2^Division of Life Sciences and Medicine, Department of Obstetrics and Gynecology, The First Affiliated Hospital of USTC, University of Science and Technology of China, Hefei, China

**Keywords:** LEEP, positive margin, influencing factors, scoring system, preoperative prediction

## Abstract

**Introduction:**

Currently, the commonly used surgical methods for cervical lesions include loop electrosurgical excision procedure (LEEP) and cold knife conization (CKC). However, the positive rate of surgical margins after LEEP is relatively high, which leads to disease recurrence and places further demand on clinical treatment. This study investigated factors related to positive margins after LEEP and established a scoring system to enhance preoperative risk assessment and surgical selection.

**Materials and Methods:**

A retrospective analysis of the clinical data of 411 patients undergoing LEEP surgery for cervical lesions in the First Affiliated Hospital of University of Science and Technology of China (USTC), from January 2016 to March 2021, was performed. Cases were divided into a negative margin group (349 cases) and a positive margin group according to postoperative pathology. In the positive group (62 cases), single-factor and multi-factor analyses screened influencing factors; a logistic and additive scoring system was established; furthermore, a ROC curve was used to evaluate scoring effectiveness.

**Results:**

The positive rate of resection margins after LEEP was 15.1%. Univariate analysis indicated a relationship to patient age, menopause, preoperative ThinPrep Cytology Test (TCT) results, lesion quadrant number under colposcopy, cervical biopsy, and the result of endocervical curettage (ECC). Multivariate analysis showed that age >35 y, menopause, preoperative TCT being high-grade squamous intraepithelial lesion (HSIL), four quadrants being involved under colposcopy, and ECC being HSIL were all independent influencing factors of positive margins after LEEP (*P* < 0.05). These were included with the above factors to establish a logistic and additive scoring system. When the logistic score was 17, the sensitivity and specificity of predicting positive margins after LEEP were 80.6 and 61.6%, respectively. When the additive score was 6, the sensitivity and specificity were 74.2 and 66.2%, respectively. Both scoring systems had good predictability (area under the curve AUC >0.75).

**Conclusions:**

This study quantified factors influencing positive margins after LEEP and established a scoring system for evaluating patients before surgery to provide a basis for individualized treatment and selection of surgical methods.

## Introduction

Cervical intraepithelial neoplasia (CIN) is closely related to cervical cancer (1). In 2014, the World Health Organization (WHO) changed the original name of CIN to squamous intraepithelial lesions and adopted a secondary classification, namely low-grade squamous intraepithelial lesion (LSIL) and high-grade squamous intraepithelial lesion (HSIL). LSIL includes CIN1 and HSIL includes CIN2 and CIN3. It is estimated that 1–2% of women worldwide suffer from CIN2-3 each year and CIN2-3 may develop into cervical cancer (2). Current treatment for CIN2-3 includes loop electrosurgical excision procedure (LEEP), cold knife conization (CKC), and cryotherapy (3); however, there is no consensus on the most effective treatment (4). Some studies show that the positive margin of cervical lesions after surgery is an important factor for recurrence and is an indicator of the quality of clinical practice (5–7). A meta-analysis indicated that the residual or recurrence risk of positive resection margins was increased compared with negative resection margins (8). Previous research also found that the risk of treatment failure with positive margins increases 5-fold (9). However, it is still unclear which factors affect positive margins after LEEP surgery.

For patients with positive margins after LEEP surgery, prediction and selection of the appropriate surgical method and scope can reduce the positive rate of postoperative resection margins and avoid residual recurrence of lesions and secondary treatment after surgery. Here, we build a scoring system to screen the risk factors associated with positive margins after LEEP, this system can provide precise and individualized treatment plans for clinicians.

## Materials and Methods

**Figure 1:**
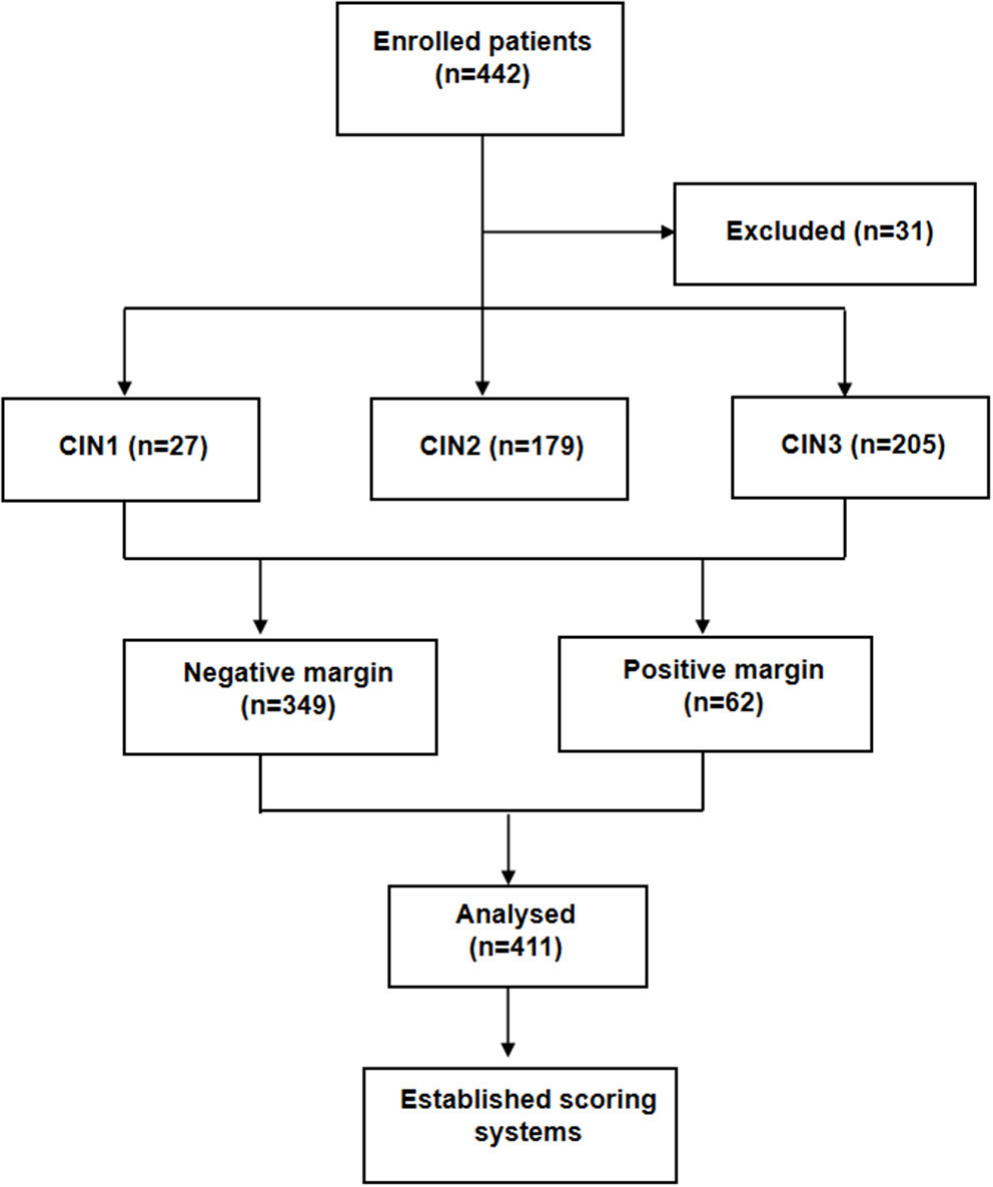
Research roadmap and basic information.

A retrospective analysis was performed on the patients undergoing LEEP surgery in the First Affiliated Hospital of University of Science and Technology of China (USTC) from January 2016 to March 2021. Inclusion criteria: pathology of the patient's preoperative biopsy confirmed cervical lesions (CIN1-3); the surgical method was LEEP; and the postoperative pathology was clear, with completely resected margins. Exclusion criteria: postoperative pathology was cervical cancer, concurrent endometrial cancer, ovarian cancer, or other malignant disease; medical records were incomplete (Figure 1).

Data collection included age, pregnancy and childbirth history, menopause, preoperative HPV testing and ThinPrep Cytology Test (TCT) results, type of transformation zone, number of lesion involvement quadrants in colposcopy, cervical biopsy pathology, gland, surgical time, and method of postoperative pathology.

### The Construction and Evaluation of Scoring Systems

Statistical methods were used to conduct single factor analysis, screen meaningful variables for multivariate logistic regression analysis, and to determine the final risk factors. A simplified model was adopted to facilitate clinical application. For the logistic scoring system: according to the results of multivariate regression analysis and the rounded value of its 10-fold regression coefficient (β ×10), each risk index was assigned. For the additive scoring system: based on the OR value of each factor in the regression analysis, a rounded integer OR value, and the sum of the OR values of each factor could predict the probability of positive surgical margins in patients with cervical lesions (10). The receiver operating characteristic (ROC) curve was used to evaluate the performance of the scoring system. The higher the AUC value, the better the predictive ability of the scoring system. The value of AUC ranges from 0.5 to 1.

### Statistical Analysis

SPSS 23.0 and MedCalc software were used for statistical analysis. To facilitate clinical application and calculation, the quantitative data were transformed into qualitative data. The single factor analysis was adopted by χ^2^ test. Meaningful predictive factors were screened according to the standard of *P* < 0.05 and included in the multivariate logistic regression analysis, based on independent influencing factors. The regression coefficient and OR value of the scoring system was established. The ROC curve and Hosmer–Lemeshow goodness-of-fit test were used to evaluate the overall effectiveness of the scoring system (11).

### Ethical Approval

In this study, medical records and treatment images of patients were retrospectively classified and exempt from informed consent. The study was approved by the Institutional Human Ethics Committee at the First Affiliated Hospital of USTC with the approval number 2021-RE-073.

## Results

### Univariate Analysis of Positive Margins After LEEP

**Figure 2:**
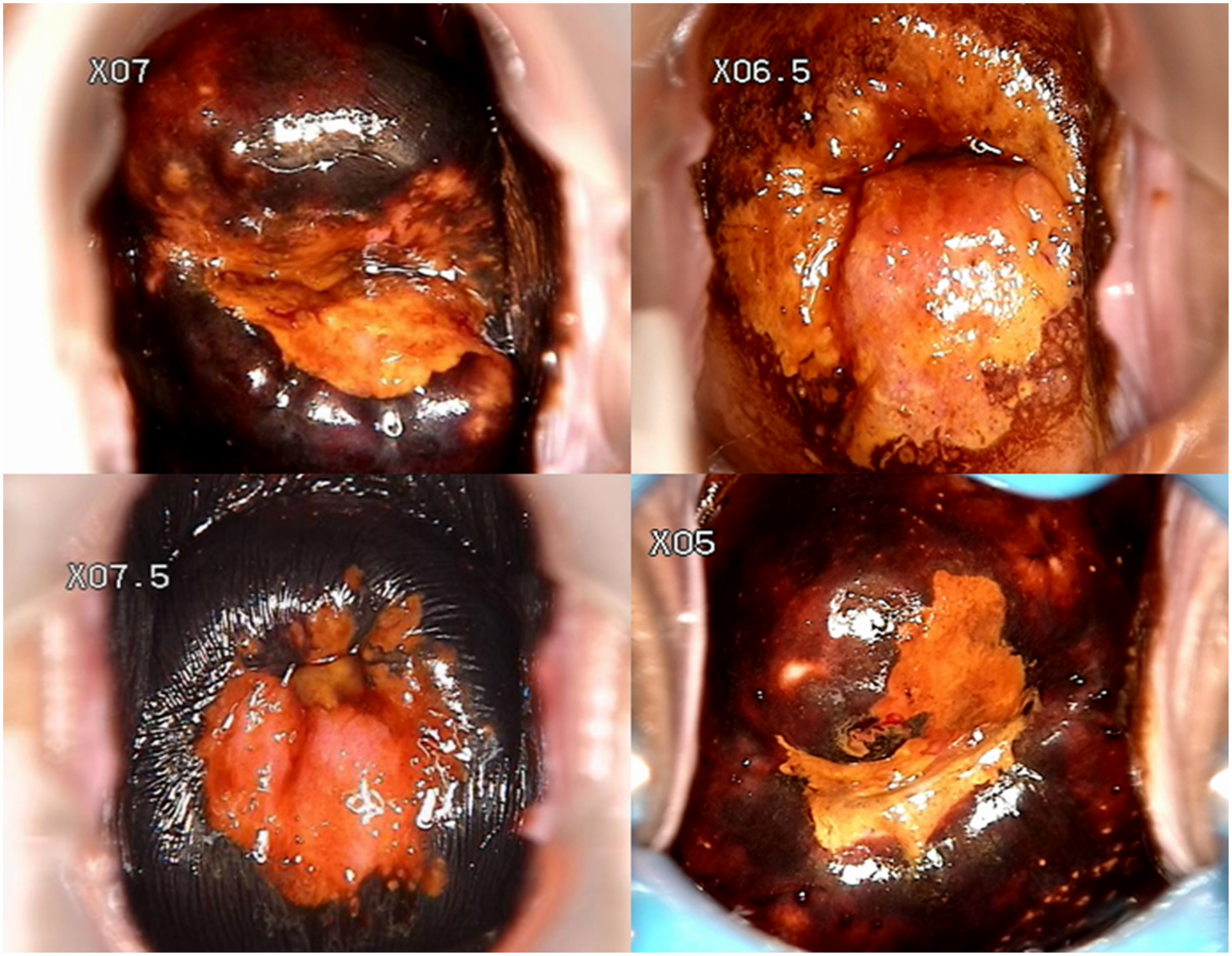
Preoperative colposcopic images of 4 patients with positive margin.

Four hundred and eleven patients who met the criteria were included in the study with an age range of 19–73 y and a median age of 39 y. There were 349 patients with negative margins and 62 patients with positive margins (Figure 2), the positive margin rate was 15.1%. Among the 60 menopausal patients, 19 (31.67%) patients had positive margins. There were a total of 125 patients with gravidity >3, of which 21 patients had positive resection margins, and 31 patients with parity >2 including 6 patients with positive resection margins. However, the number of gravidity and parity were not statistically significant. Moreover, a total of 73 patients had preoperative TCT with HSIL, including 19 (26.03%) patients with positive margins. Furthermore, there were 90 patients with type 1 transformation zone, 91 patients with type 2 transformation zone, and 230 patients with type 3 transformation zone.

Preoperative cervical biopsy revealed 27 cases of CIN1, 179 cases of CIN2, and 205 cases of CIN3. One hundred and sixty-six patients were infected with HPV16/18, of which 24 patients had positive resection margins. There were 238 patients with other high-risk HPV infections. We found that 155 patients had cervical lesions involving the glands, including 27 patients with positive margins, but no significant difference of gland involvement was observed. The results of endocervical curettage indicated 15 cases of HSIL, and 40% of HSIL patients had positive resection margins. Univariate analysis suggested that patient age, menopause, preoperative TCT, quadrant number of lesions under colposcopy, and the results of endocervical curettage (ECC) were significantly different between the two groups (*P* <0.05; Table 1).

**Table 1:** Single factor analysis of positive margins after LEEP.

**Variable**	**Negative margin (*n* = 349)**	**Positive margin (*n* = 62)**	***χ^2^***	***P***
Age(years)				
≤35	128	11	8.433	**0.004**
>35	221	51		
Menopause				
NO	308	43	15.08	**<0.001**
YES	41	19		
Gravidity (times)				
≤3	245	41	0.412	0.521
>3	104	21		
Parity (times)				
≤2	324	56	0.477	0.49
>2	25	6		
Preoperative TCT^a^				
Non-HSIL	294	41	9.085	**0.003**
HSIL	54	19		
HPV type^b^				
Type 16 and/or 18	142	24	0.171	0.68
Other high-risk HPV types	207	31		
Transformation zone				
Type 1	73	17	4.011	0.135
Type 2	83	8		
Type 3	193	37		
Number of quadrants involved in lesions under colposcopy				
≤3	212	22	13.702	**<0.001**
>3	137	40		
Cervical biopsy				
≤CIN2	181	25	2.804	0.094
>CIN2	168	37		
Gland involvement				
N0	221	35	1.058^a^	0.304
YES	128	27		
Endocervical curettage				
Non-HSIL	340	56	7.544	**0.006**
HSIL	9	6		

*Missing data are not included in statistics*.

a *Three cases had missing data*.

b *Seven cases had missing data*.

*P < 0.05 are indicated with bold characters*.

### Multivariate Logistic Regression Analysis of Positive Resection Margin

Multivariate regression analysis showed that the index, including age >35 y (β: 0.823; OR: 2.278; 95% CI: 1.067–4.863), menopausal (β:1.32; OR: 3.745; 95% CI: 1.771–7.919), preoperative TCT with HSIL (β:0.942; OR: 2.566; 95% CI: 1.279–5.145), four quadrants under colposcopy (β:1.541; OR: 4.671; 95% CI: 2.393–9.114), and HSIL of ECC (β:1.64; OR: 5.154; 95% CI: 1.527–17.404), were independent risk factors for positive postoperative margins (*P* < 0.05; Table 2).

**Table 2:** Multivariate analysis.

**Variable**	**β**	**SE**	**Wald**	***P***	**OR**	**95% CI**
Age (>35 years)	0.823	0.387	4.524	0.033	2.278	1.067~4.863
Menopause (Yes)	1.32	0.382	11.942	0.001	3.745	1.771~7.919
Preoperative TCT(HSIL)	0.942	0.355	7.044	0.008	2.566	1.279~5.145
Number of quadrants involved in lesions under colposcopy (>3)	1.541	0.341	20.416	<0.001	4.671	2.393~9.114
Endocervical curettage (HSIL)	1.64	0.621	6.977	0.008	5.154	1.527~17.404

*TCT, ThinPrep Cytology Test; HSIL, high grade squamous intraepithelial lesion. P values < 0.05 are indicated with bold characters*.

### The Establishment of Scoring Systems

According to the regression coefficient (β) and OR value of the multivariate logistic regression equation variables, two systems were established to predict whether the margins were positive after LEEP surgery. The logistic scoring system scored from 0 to 61 points, and the additive scoring system scored from 0 to 19 points (Table 3).

**Table 3:** Scoring systems.

**Variable**	**Logistic**	**Additive**
Age (>35 years)	8	2
Menopause (Yes)	13	4
Preoperative TCT (HSIL)	9	3
Number of quadrants involved in lesions under colposcopy (>3)	15	5
Endocervical curettage (HSIL)	16	5

### Evaluation of the Scoring Systems

**Figure 3:**
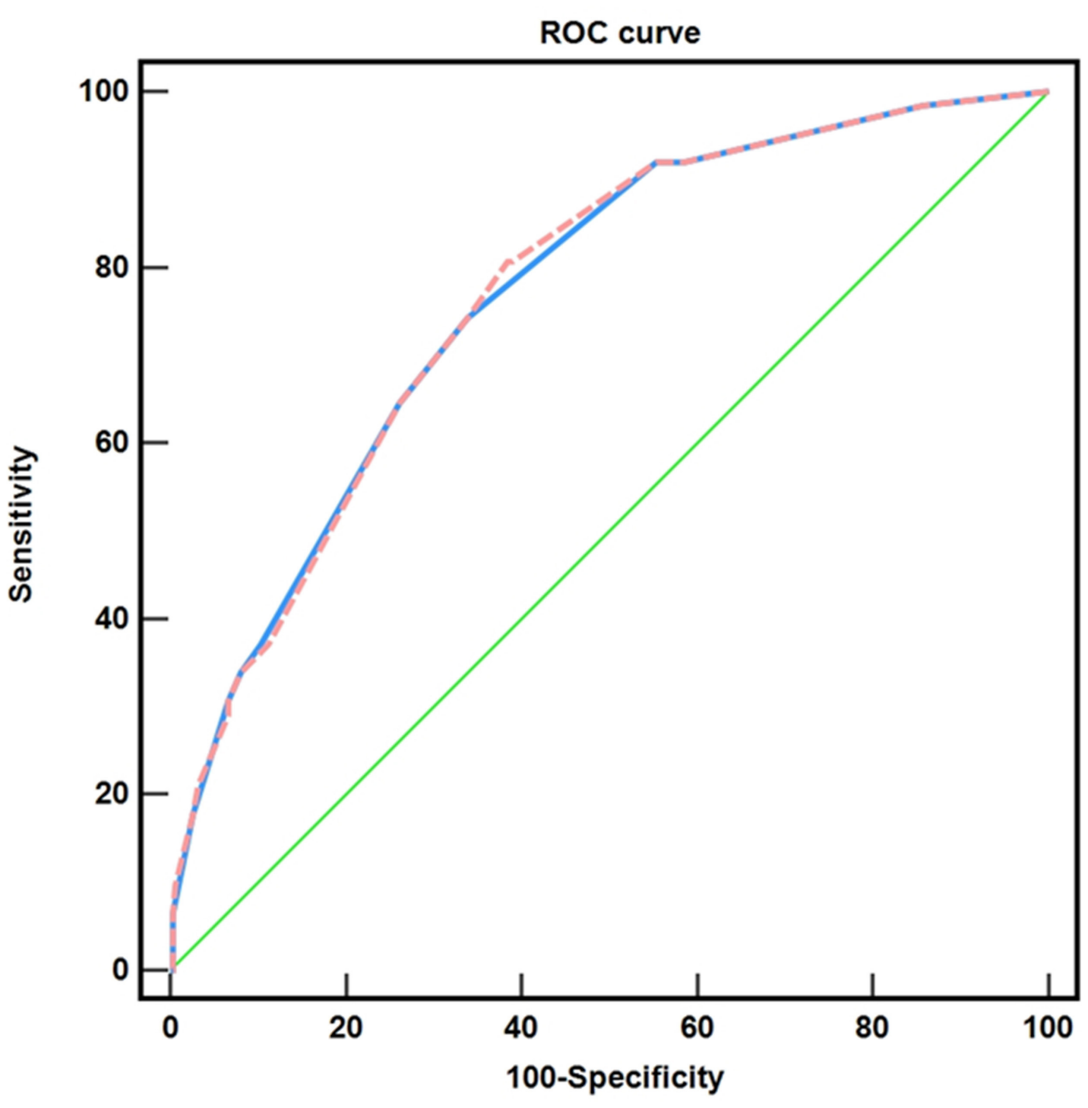
Comparison of the two risk scoring systems. (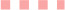) the logistic scoring system, AUC: 0.769; (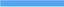) the additive scoring system, AUC: 0.768; (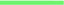) reference line.

Based on the investigated factors related to positive margins after LEEP, we established a scoring system to enhance preoperative risk assessment and surgical selection. We found that when the logistic score was 17, the sensitivity and specificity were 80.6 and 61.6%, respectively; the AUC was 0.769, 95% CI: 0.709–0.829; the Hosmer–Lemeshow goodness of fit test, χ2 = 2.433, *P* > 0.05. Furthermore, when the additive score was 6, the sensitivity and specificity were 74.2 and 66.2%, respectively; the AUC was 0.768, 95% CI: 0.707–0.828; the Hosmer–Lemeshow goodness of fit test, χ2 = 1.885, *P* > 0.05 (Figure 3).

## Discussion

With the promotion of cervical cancer screening and the improvement of medical awareness, the population of patients with cervical precancerous lesions is increasing. It is particularly important to predict the development of the disease or the adverse consequences associated with treatment in the early stage and to provide effective evidence for patients' personalized treatment (12, 13). Previously, we established the risk scoring system for lymph node metastasis of early endometrial cancer and the scoring system for postoperative decision-making of advanced cervical cancer, which were applied in clinic (10). At present, there is no uniform standard for the selection of cervical lesions. The 2019 American Society of Colposcopy and Cervical Pathology (ASCCP) pointed out that resection was better than ablation therapy for cervical HSIL (14), while WHO opposed cold knife conization (CKC) as the first choice (3). The expert consensus of the Chinese Society of Eugenics Science for Colposcopy and Cervical Pathology (CSCCP) also made no relevant recommendations on the surgical methods of cervical lesions. The choice of surgical method mostly depends on the subjective judgment of the surgeon. Thus, we analyzed the factors influencing positive margins after LEEP and established a scoring system for evaluating patients before surgery and to provide a basis for individualized treatment and selection of surgical methods.

Compared to LEEP, CKC is associated with increased postoperative bleeding, infection, premature birth, and other adverse events, but the postoperative recurrence rate is lower (15). Studies show that CKC reduces the risk of cervical residual disease, and positive margins in women undergoing LEEP surgery increase 2-fold compared with CKC (4, 16). Although a positive margin after surgery does not imply a persistent postoperative lesion, the follow-up treatment requires a comprehensive evaluation for individualized treatment (17, 18). Although the US and European guidelines recommend repeat surgery for patients with positive margins after minimally invasive cervical cancer resection, no consensus has been reached on the further treatment of positive margins for patients with CIN2 and CIN3. The subsequent treatment mainly includes cytological and colposcopic follow-up, conization, or hysterectomy (19, 20). Additionally, regular follow-up may make the patient anxious, and missed diagnosis or repeat surgeries also lead to related surgical complications. Therefore, the choice of the first surgical method is very important. The purpose of this study was to construct a scoring system to evaluate the risk population with positive margins after LEEP surgery.

**Figure 4:**
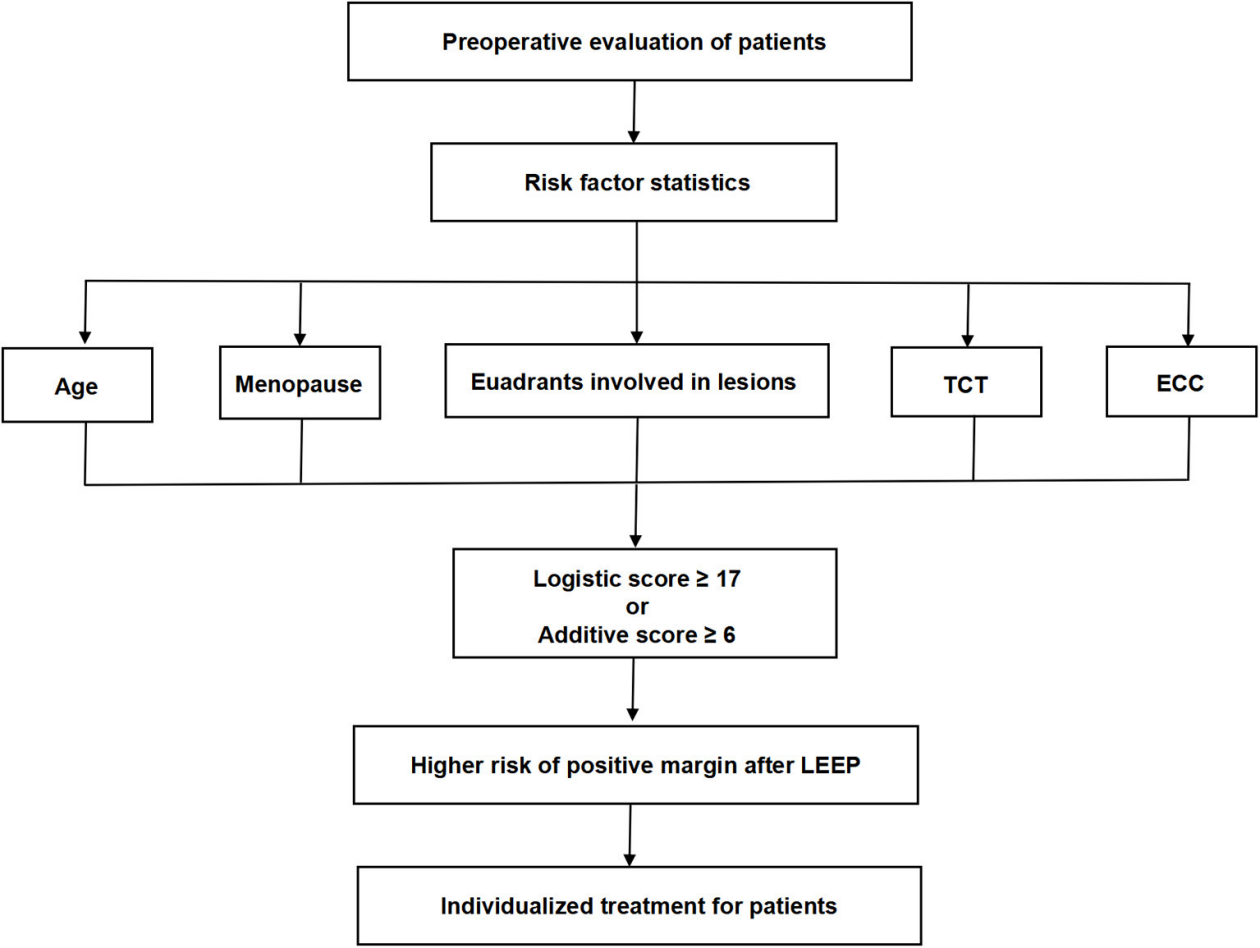
Application process of the scoring system for preoperative evaluation.

In this study, we found that when the logistic score was 17, the sensitivity and specificity were 80.6 and 61.6%. Furthermore, when the additive score was 6, the sensitivity and specificity were 74.2 and 66.2%. Thus, when patients had two or more independent risk factors, the risk of positive margins after LEEP surgery rose sharply. For such patients, clinicians need to carefully consider the choice of surgical method (Figure 4). Independent risk factors for positive margins included age >35 y, menopause, HSIL in preoperative TCT or ECC, and colposcopy lesions involving four quadrants. Xiang found that the positive rate of surgical margins was 3.7% for women who were never delivered, while it increased to 7.8% for women with history of childbirth (21). Our study found that multiple pregnancy (number of pregnancies >3) and prolific birth (parity >2) were not related to positive surgical margins. Previous reports showed that the risk of residual LSIL lesions in 25 y and 40 y patients after LEEP were 20 and 30%, and the incidence of HSIL in women <35 y is 2-fold of women >35 y (6.5 vs. 3.7%) (22, 23). Liu described the age of high incidence of HSIL/LSIL as 35–49 y (24). Here, the age of 35 y was also taken as the boundary to separate the age of menopause as this age group is of childbearing age and had one of the highest incidences of cervical lesions. We also found that age >35 y was a risk factor for positive margins after LEEP surgery (*P* = 0.033), which is consistent with the previous results (25). Previous studies showed that menopause was a risk factor for positive surgical margins (26, 27), and our research also confirmed these findings. The cervix of postmenopausal women atrophies and the squamous column junction moves inward, which increases the difficulty of lesion detection and operation.

As the main screening method for cervical cancer, TCT combined with HPV examination can identify most cervical lesions (28) and their comprehensive detection effect is comparable to histopathological examination. Some studies showed that the sensitivity, specificity and positive prediction of TCT were 78.3, 77.9 and 73.3%, respectively, which was higher than the HPV test (29). In this study, compared with other types of HPV, HPV16/18 infection had no effect on the positive margin, which is consistent with a previous study (26). However, postoperative TCT with HSIL is a risk factor of positive resection margin. This may be because the TCT results are related to the degree of disease. This finding also suggested that the positive margin after LEEP was not correlated with the type of HPV infection, but was dependent on the degree of disease progression. Under clinical conditions including: an unsatisfactory colposcopy; atypical squamous cells of undetermined significance (ASCUS) in cytology and positive HPV but without visible lesion under the colposcopy; ≥LSIL in cytology and no lesion under colposcopy; HSIL cytology; atypical glandular cells (AGC); or a suspected invasive disease, ECC was suggested as the prioritizable method (30). In our study, we also found that the HSIL of ECC was a risk factor for positive margins, which was supported by the previous study that the risk of surgical margins with positive ECC was increased (31). At present, TCT and ECC are mainly used for cervical cancer screening and diagnosis, but their impact on prognosis is often ignored clinically. Patients with higher cytological atypia also have a higher probability of positive margins and residual lesions (25). Here, our study emphasizes the importance of preoperative cytological results for patient evaluation. This study also showed that preoperative TCT and ECC results of HSIL were risk factors for positive margin, but cervical biopsy of CIN3 were not related. This reflects the importance of cytological diagnosis for the treatment and prognosis of cervical lesions. TCT is the detection of cervical transformation area and a small number of intracervical cells, but intracervical cell analysis depends on ECC. It is undeniable that colposcopy images and biopsy histopathology are important for the diagnosis of cervical lesions, but the selection of biopsy location and the determination of biopsy tissue size are lack of standardization, which often affects the disease diagnosis of patients (32). For example, Lang pointed out that the pathological grade of ECC was higher than that of tissue biopsy (33). Thus, the combined application of cytopathology and histopathology not only makes the disease diagnosis more accurate, but also provides a basis for the selection of disease treatment and prognosis. Furthermore, postoperative TCT combined with HPV detection is a common close monitoring method for patients with positive margin. Postoperative HPV infection and abnormal TCT results are closely related to disease recurrence. Postoperative TCT can predict disease recurrence in patients with positive margin, but cannot infer lesion residue (16), so the choice of initial treatment is also important for patients. Additionally, a larger lesion area was a risk factor for positive surgical margins and more extensive changes in high-grade disease were closely related to incomplete resection (34). The current study found that when four quadrants were involved during colposcopy, the positive rate of resection margin after LEEP was as high as 22.60%, while the range of other lesions was only 9.40%. The large lesion range increases the operation difficulty of the operator and LEEP is more difficult for the complete resection of large-scale cervical lesions. Some studies have pointed out that the experience and professionalism of doctors are related to the positive rate of surgical margin, and inexperienced surgeons may increase the positive rate of surgical margin (26, 35). In our study, all surgeons had been engaged in colposcopy diagnosis and treatment of cervical lesions for many years and had rich clinical experiences and theoretical knowledge to avoid the influence of surgeons on surgical margins.

## Conclusions

Here, we preliminarily constructed a scoring system for positive LEEP postoperative cutting edge, namely the logistic scoring system and the additive scoring system. The AUC was 0.769 (0.709–0.829) and 0.768 (0.707–0.828), respectively, indicating that the scoring system was effective. This system is suitable for cervical intraepithelial neoplasia. Nevertheless, it is still necessary to expand the sample size to further test the scoring system so that patients with cervical lesions can be evaluated before surgery according to specific clinical guidelines.

## Data Availability Statement

The original contributions presented in the study are included in the article/supplementary material, further inquiries can be directed to the corresponding authors.

## Ethics Statement

The studies involving human participants were reviewed and approved by the Institutional Human Ethics Committee at the First Affiliated Hospital of the University of USTC with the approval number 2021-RE-073. Written informed consent for participation was not required for this study in accordance with the national legislation and the institutional requirements.

## Author Contributions

WZ and ZC participated in the design of the study. MZ and MY planned the analyses. MZ managed the data, performed the analysis, and wrote the first draft of the manuscript. WZ gave input to all phases of the study, contributed to critical revision of the manuscript, and approved the final version. All authors contributed to the article and approved the submitted version.

## Funding

This work was supported by the National Natural Science Foundation of China (82172774), the Fundamental Research Funds for the Central Universities (WK9110000150), and the University Synergy Innovation Program of Anhui Province (GXXT-2019-044).

## Conflict of Interest

The authors declare that the research was conducted in the absence of any commercial or financial relationships that could be construed as a potential conflict of interest.

## Publisher's Note

All claims expressed in this article are solely those of the authors and do not necessarily represent those of their affiliated organizations, or those of the publisher, the editors and the reviewers. Any product that may be evaluated in this article, or claim that may be made by its manufacturer, is not guaranteed or endorsed by the publisher.

## Abbreviations

ASCCP: American Society of Colposcopy and Cervical Pathology
AUC: Area under the curve
CIN: Cervical intraepithelial neoplasia
CSCCP: Chinese Society of Eugenics Science for Colposcopy and Cervical Pathology
CKC: Cold knife conization
ECC: Endocervical curettage
HSIL: High-grade squamous intraepithelial lesion
LEEP: Loop electrosurgical excision procedure
LSIL: Low-grade squamous intraepithelial lesion
ROC: Receiver operating characteristic
TCT: ThinPrep Cytology Test
OR: Odds ratio
USTC: University of Science and Technology of China
WHO: World Health Organization.
